# Vasoprotective effects of human CD34+ cells: towards clinical applications

**DOI:** 10.1186/1479-5876-7-66

**Published:** 2009-07-29

**Authors:** Thomas J Kiernan, Barry A Boilson, Tyra A Witt, Allan B Dietz, Amir Lerman, Robert D Simari

**Affiliations:** 1 Division of Cardiovascular Diseases, Mayo Clinic, 200 First Street SW, Rochester, MN 55905, USA; 2 Division of Transfusion Medicine, Mayo Clinic, 200 First Street SW, Rochester, MN 55905, USA

## Abstract

**Background:**

The development of cell-based therapeutics for humans requires preclinical testing in animal models. The use of autologous animal products fails to address the efficacy of similar products derived from humans. We used a novel immunodeficient rat carotid injury model in order to determine whether human cells could improve vascular remodelling following acute injury.

**Methods:**

Human CD34+ cells were separated from peripheral buffy coats using automatic magnetic cell separation. Carotid arterial injury was performed in male Sprague-Dawley nude rats using a 2F Fogarty balloon catheter. Freshly harvested CD34+ cells or saline alone was administered locally for 20 minutes by endoluminal instillation. Structural and functional analysis of the arteries was performed 28 days later.

**Results:**

Morphometric analysis demonstrated that human CD34+ cell delivery was associated with a significant reduction in intimal formation 4 weeks following balloon injury as compared with saline (I/M ratio 0.79 ± 0.18, and 1.71 ± 0.18 for CD34, and saline-treated vessels, respectively P < 0.05). Vasoreactivity studies showed that maximal relaxation of vessel rings from human CD34+ treated animals was significantly enhanced compared with saline-treated counterparts (74.1 ± 10.2, and 36.8 ± 12.1% relaxation for CD34+ cells and saline, respectively, P < 0.05)

**Conclusion:**

Delivery of human CD34+ cells limits neointima formation and improves arterial reactivity after vascular injury. These studies advance the concept of cell delivery to effect vascular remodeling toward a potential human cellular product.

## Background

Cellular therapies hold great promise for the treatment of human disease. The development of cell-based therapeutics for humans requires preclinical testing in animal models. There are inherent limitations to the use of autologous animal products for preclinical testing. First, the use of autologous animal products fails to address the specific efficacy of the intended human product. Second, immunophenotyping of animal products may be limited by a lack of reagents which are available for use in humans and thus fail to predicate human results. To overcome these limitations and in order to develop novel human cellular products, immunodeficient animals may be used to test the delivery of these products.

We and others have demonstrated the vasculoprotective effects of local delivery of circulation and adipose-derived cells with an endothelial phenotype following acute vascular injury [1-4]. These effects include a reduction in neointimal formation and improvement in vascular reactivity. These studies suggest that cell delivery may improve large vessel healing which might be extrapolated to clinical scenarios such as post-angioplasty or stenting. However, the translational potential of these studies has been hindered by two important issues. First, the cells have been cultured under variable conditions prior to delivery [1,2]. Second, rabbit-specific reagents that define circulating precursors are limited. Thus, identification of a circulating cell capable of these vasoprotective effects would be an advance.

CD34 is a hematopoietic progenitor cell marker. In a landmark publication by Asahara in 1997, bone marrow derived cells expressing CD34 were demonstrated to differentiate *ex vivo *to an endothelial phenotype [5]. The function of CD34 is uncertain, but it is thought to be a cell to cell adhesion molecule that anchors hematopoeitic progenitor cells to the bone marrow stroma and also facilitates their interaction with other stromal cells. Interestingly, it is also known that there is a complex interaction between bone marrow derived progenitor cells (hematopoetic progenitor cells, HPCs) and microvascular endothelial cells in bone marrow. Endothelial cells appear to regulate the trafficking and release of HPCs from bone marrow [6]. CD34 is also expressed on microvascular endothelial cells, and this shared antigen expression between microvascular endothelium and hematopoietic progenitors is also strongly supportive of a shared embryological origin and that hematopoiesis and vasculogenesis are linked in the embryo. The ability of circulating CD34+ cells to adapt an endothelial phenotype is well established [5]. As such, we aimed to test the hypothesis that delivery of human CD34+ cells would be vasculoprotective. To do so, we developed a model of acute carotid artery injury in an immunodeficient rat model.

## Methods

### Isolation and selection of human CD34+ cells from peripheral blood

Leukocyte filter eluates (10 mls) of human whole blood were obtained from normal donors after leukophaeresis [7]. Human whole blood samples were obtained from healthy volunteers after approval from the Mayo Clinic Institutional Review Board Approval. The cells were incubated with anti-CD34-conjugated superparamagnetic microbeads (CD34 Isolation kit; Miltenyi Biotec), washed, and processed to obtain purified CD34 cells. FACS was also performed on freshly immunoselected CD34 cells to determine their phenotypic profile and purity.

### Flow cytometry

Purified cells were counted and re-suspended in seven 100 μL aliquots of PBS for FACS analysis, each containing approximately 10^5 ^cells. After addition of Fc receptor blocking antibody (Miltenyi Biotec) to each tube, cells were incubated with fluorochrome-conjugated antibodies to CD34 (FITC), CD45 (PerCP) (both from BD biosciences), CD133 (PE) (Miltenyi Biotec), and VEGFR2 (APC) (R&D Systems). Murine IgG_1 _(R&D Systems) conjugated to Alexa 488, PE (Molecular Probes), and Rat anti-mouse PerCP (BD Biosciences) was used as isotype controls as well as IgG_1_-APC from BD Biosciences.

### Carotid injury model in immunodeficient rats

All animal procedures were approved by the Mayo Clinic Institutional Animal Care and Use Committee. Immunodeficient rats (Sprague-Dawley) were housed at constant room temperature (24 ± 1°C) and humidity (60 ± 3%). The athymic nude mutant rat (Hsd:RH-Foxn1^rnu) represents a well-established research model that has already made a substantial contribution to many scientific disciplines, such as immunology and cancer research. The *rnu *allele on chromosome 10 is an autosomal recessive mutation associated with hairlessness and thymic aplasia. The thymus-dependent lymph node areas are depleted of lymphocytes (T-cells). The animals are phenotypically hairless and have rudimentary thymic tissue present. Male e rats (3 to 4 months old weighing 350 to 400 g) were anesthetized with an intramuscular injection of ketamine 50 mg/kg, xylazine 10 mg/kg, and acepromazine 1 mg/kg. Under general anaesthesia and by using an operating microscope, a midline incision was made in the neck to expose the left external carotid artery. A 2F Fogarty balloon embolectomy catheter (Baxter) was introduced into the left external carotid artery and advanced through the common carotid artery to the aortic arch. The balloon was inflated with saline (0.02 ml) until a slight resistance was felt and then was rotated while pulling it back through the common carotid artery to denude the vessel of endothelium. This procedure was repeated two more times (total of three passes), and then the catheter was removed. Immediately after catheter withdrawal, residual material was removed and 200 μl of saline with freshly selected CD34+ cells and saline alone was administered locally by intra-vascular instillation for 20 minutes through a 24G catheter. The external carotid was ligated with a 6-0 silk suture and the blood flow restored by removing the clips at the common and internal carotid arteries. After inspection to ascertain adequate pulsation of the common carotid artery, the surgical incision was closed, and the rats were allowed to recover from anaesthesia in a humidified and warmed chamber for 2 to 4 hours. The animals were euthanized with an overdose of pentobarbital (200 mg/kg) 28 days after balloon injury, and the carotid arteries were collected for molecular, mechanical, and histological analyses.

### Cell tracking Studies

In order to track the fate of delivered cells, human CD34+ cells were labelled with CM-DiI (1 μg/ml), a fluorescent membrane dye (Molecular Probes), and resuspended in 200 μl saline for subsequent administration. Animals were euthanized after 4 weeks with an overdose of pentobarbital sodium. Both carotids were excised, embedded in OCT (Tissue-Tek), and immersed in 2-methylbutane cooled by liquid nitrogen. Mounted 5 μm sections were examined under fluorescence microscopy for detection of CM-DiI-labeled cells.

### Effects of cell delivery on vascular form and function

Immunodeficient rats were assigned to 3 groups (n = 8 per group) to determine vasoreactivity and development of neointima formation at 4 weeks after balloon injury. Group 1 rats received no balloon injury and served as uninjured controls. Group 2 rats underwent balloon catheter injury to the left common carotid artery, received human CD34 cells as defined above, and were sacrificed at 4 weeks after balloon injury. Group 3 rats underwent balloon catheter injury to the left common carotid artery, received normal saline, and were sacrificed at 4 weeks after balloon injury.

### Arterial vasoreactivity

Four weeks after balloon injury and local CD34+ cells or saline delivery, animals were euthanized and carotids immediately immersed in cold Krebs solution. Arterial rings ~3 mm in length (3 per artery) were carefully dissected from the surrounding adipose tissue under a microscope with great care taken to protect the endothelium. The carotid rings were then connected to isometric force displacement transducers and suspended in organ chambers filled with 25 ml of Krebs (94% O_2_, 6% CO_2_) solution. Rings were equilibrated for 1 hour at 37°C and then incrementally stretched to 2 g. Viability and maximum contraction was determined with 60 mM KCl. After 3 washes with Krebs solution and further equilibration, arteries were precontracted with phenylephrine in a titrated fashion to achieve ~80% stable maximal contraction. To study endothelium dependent relaxation, acetylcholine (10^-9 ^to 10^-5 ^M) was added to the organ bath in a cumulative manner. Following 3 further washes and equilibration, the arteries were recontracted, and viability was confirmed by assessment of endothelium independent responses to sodium nitroprusside, an exogenous NO donor.

### Morphometric analysis

The carotid arteries were perfusion-fixed at a constant physiological pressure of 125 mm Hg with 4% paraformaldehyde. The carotid arteries were carefully stripped of adventitia and excised between the origin at the aorta and the carotid bifurcation. The proximal segment (0.3 cm) of the denuded arteries was removed and fixed in 4% paraformaldehyde for 12 hours before being embedded in paraffin and used for morphometric analysis. The cross sections (5 μm) of carotid artery were generated at 200 μm intervals, paired slides being then stained with LELVG or H&E for morphometric analysis. The first three slides (400 μm apart) were analyzed to define the effects on neointimal formation. Endoluminal, internal elastic laminar and external elastic laminar borders were manually traced, digitally measured, and analyzed using software (Image ProPlus) to calculate intimal and medial areas. Because native media thickness is variable (reflecting the diameter of the artery), it was used to index the area of neointima resulting from balloon injury. Accordingly, neointimal thickness was assessed in terms of intima to media area ratios.

### Statistical analysis

Vasoreactivity data were analyzed with ANOVA for repeated measures; direct pair wise comparisons between groups were made with Scheffe's t-test. Intima/Media ratios were compared with unpaired t-tests. A value of P < 0.05 was considered to be statistically significant. Data are presented as mean ± SEM.

## Results and discussion

### Isolation and characterization of human CD34+ cells

**Figure 1:**
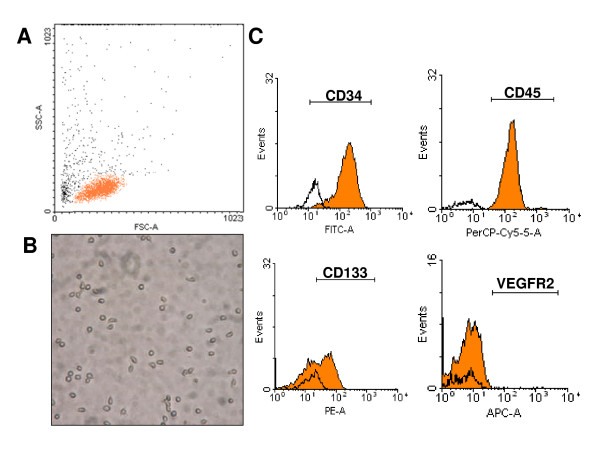
**Characterization of human CD34+ cells**. **A**. Scatter analysis reveals a low side scatter and low to intermediate forward scatter population in keeping with small round cells, as shown in (**B**) photomicrograph (200×). **C**. FACS analysis of isolated cells. CD34+ cells express CD45dim and CD133 but not VEGFR2.

Human CD34+ cells (1 to 3 × 10^6 ^CD34+ cells) were obtained from normal human donors using two sequential positive magnetic automated cell separations (MACS) immediately upon receipt of blood sample. Freshly isolated CD34+ cells from blood (purity 87 ± 13%) uniformly expressed CD45_dim _while 61 ± 9% of cells expressed CD133 and less than 1% of CD34+ cells were positive for VEGFR2 (Figure 1).

### Tracking of delivered human CD34+ cells

**Figure 2:**
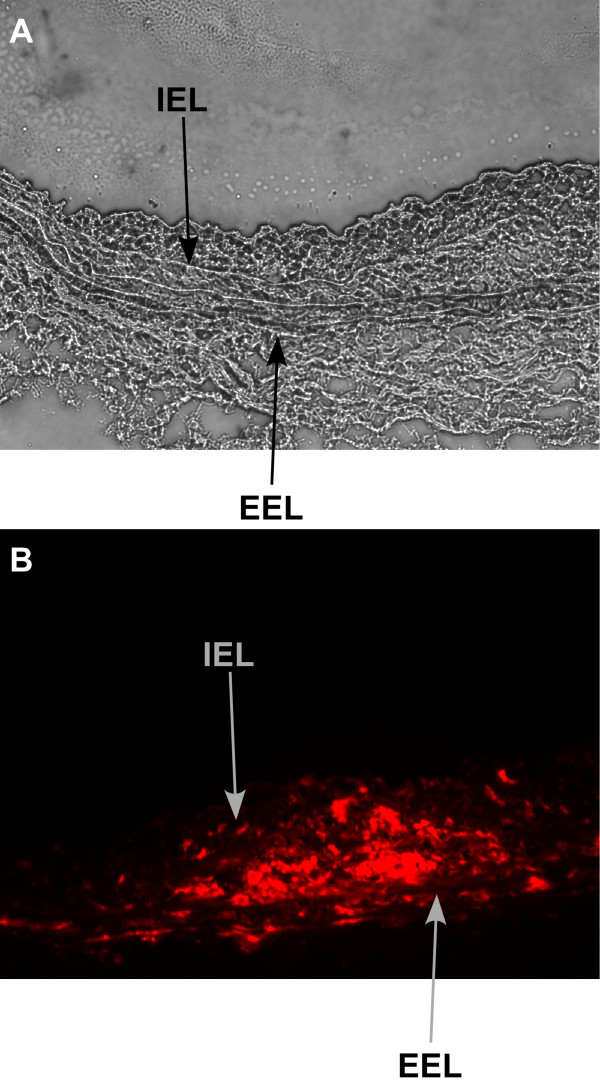
**Tracking of delivered cells**. Light microscopy cross section (20×) showing neointima formation in immunodeficient rat carotid 4 weeks after balloon injury (**A**). CM-Dil-labeled human CD34+ cells stain red under fluorescent microscope (20×) within intima and media of carotid 4 weeks after balloon injury (**B**). IEL = Internal elastic lamina, EEL = external elastic lamina.

To determine whether delivery of cells resulted in any cell retention for the 4 weeks following delivery, carotid sections were examined under fluorescence microscopy for detection of CM-DiI-labeled cells. Specific red fluorescence identified the presence of labeled human CD34+ cells within the neointima, media, and adventitia of injured segments. No labeled cells were identified in uninjured control arteries. In animals receiving human CD34+ cells, only 12.5% of carotid sections demonstrated fluorescent luminal endothelial cells at 4 weeks. Labeled cells were seen in the media (Figure 2) but also in the neointima and adventitia under fluorescent microscopy. This finding is very consistent with previous findings in circulation-derived cells [1] and suggests a paracrine mechanism for these effects.

### Vasculoprotective effects of peripheral human CD34+ cells

**Figure 3:**
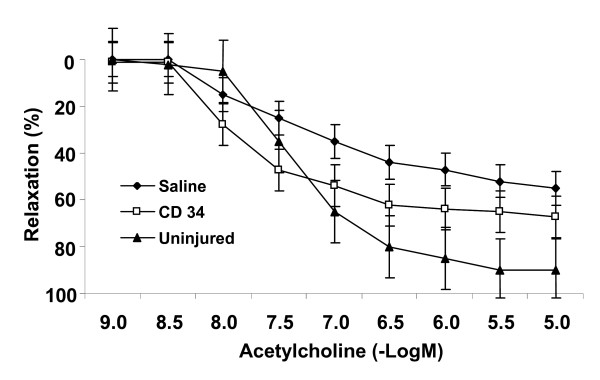
**Cell delivery improves vasoreactivity**. Human CD34+ cell delivery improves endothelium-dependent vasoreactivity after arterial injury. Carotid rings from CD34+ cell treated rats (open squares) show markedly enhanced vasoreactivity to acetylcholine 4 weeks after injury compared to saline controls (diamonds)(*P *< 0.05 for CD34+ cells vs. saline). However, uninjured left carotid arteries retained the largest relaxation responses (*P *< 0.05, vs. CD34+ treated rings). Values are means ± SE. n = 8/group.

Four weeks after balloon injury and local delivery of CD34+ cells or saline, animals were euthanized and carotids immediately immersed in cold Krebs solution. Following pre-contraction with phenylephrine in an organ chamber, relaxation in response to incremental doses of acetylcholine was assessed (Figure 3). Maximal relaxation of vessel rings from human CD34+ treated animals was significantly enhanced compared with saline-treated counterparts (74.1 ± 10.2 and 36.8 ± 12.1% relaxation for CD34+ cells and saline, respectively, *P *< 0.05 for CD34+ cells vs. saline). The concentration (-Log M) of acetylcholine required to achieve 25% of maximal relaxation (EC_25_) was 7.19 ± 0.04 in CD34 treated animals compared with 5.38 ± 0.06 in saline treated animals (p < 0.005). Although the data clearly demonstrates that CD34+ cell delivery enhanced endothelium dependent vasorelaxation, responses did not achieve those of uninjured vessels which retained the largest responses to acetylcholine (p < 0.05 for maximal relaxation and EC_50 _compared with CD34 treatment).

**Figure 4:**
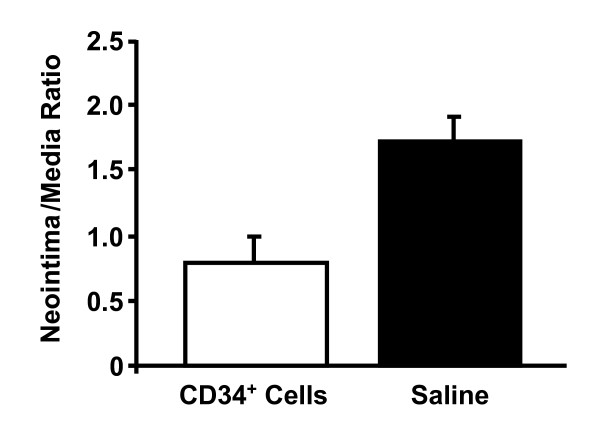
**Cell delivery reduces neointimal formation**. Local delivery of human CD34+ cells reduces neointimal formation after balloon injury. Significant attenuation of intima-to-media ratio in CD34+ treated vessels compared with saline treated control groups 4 weeks after injury (*P *< 0.05 for CD34+ cells vs. saline). n = 8/group.

Morphometric analysis demonstrated that human CD34+ cell delivery was associated with a significant reduction in neointimal formation 4 weeks following balloon injury as compared with saline. Intima-to-media ratios were 0.79 ± 0.18, and 1.71 ± 0.18 for CD34, and saline-treated vessels, respectively (P < 0.05 for CD34 vs. saline treated vessels) (Figure 4). This suggests that, in addition to improving endothelium-dependent relaxation, local delivery of CD34+ cells also attenuated neointimal formation after arterial injury in this immunodeficient rat model.

### Why CD34 + cells?

Endothelial progenitor cells (EPCs) are the most studied vascular progenitors [8]. New understandings of the inherent role of circulating cells, including precursor cells, in postnatal neovascularization have presented novel therapeutic opportunities. Studied applications of endothelial-lineage cell therapy have demonstrated enhancement of new capillary formation in ischemic tissue (therapeutic vasculogenesis) and generation of an anti-thrombogenic luminal surfaces in prosthetic grafts [9-13].

The current study tested whether specifically selected fresh human CD34+ cells without culture modification may have an applied role in modulating the vascular response to balloon injury. Unfortunately, no single definition of vascular progenitor cells exists, and it is unknown which is the best antigenic profile to identify progenitor cells linked to vascular and endothelial disease. Additionally, it is unclear as to what defines the best cells for vasculoprotective delivery. Performance of these studies necessitated the use of human reagents and an immunodeficient model. Therefore, this current study using freshly derived cells of surface antigens, represents a valid alternative of cellular therapy for vascular disease being time-saving, inexpensive, precise, and reproducible. Also, this reagent has been used extensively in humans for transplantation with an excellent safety profile.

The finding of delivered cells over a small proportion of the luminal surface suggests direct but incomplete participation of CD34+ cells in endothelial re-surfacing. Although the proportion may have been underestimated due to loss of fluorescence with cell division, it should not have been to such an extent as seen in our study. Thus, indirect mechanisms may also be involved. CD34+ cell incorporation may alter the kinetics of the denuded surface to induce proliferation of neighboring resident endothelium or recruit additional circulating precursors. In support of this possibility, it has been shown that BM-endothelial lineage cells express angiogenic ligands and cytokines [14,15] and induce proliferation of preexisting vasculature in the vicinity of myocardial infarcts [16].

The margin by which CD34+ cell delivery improved endothelial-dependent vasoreactivity is an important feature of this study. The effect is likely to be mediated at least in part by accelerated re-endothelialization. However, non-luminally located cells (as were also found in this study) could additionally influence vascular reactivity through paracrine mechanisms including the release of nitric oxide (NO) into the surrounding milieu. Indeed, adenoviral gene transfer of eNOS to the adventitia has been shown to improve NO production and vasoreactivity even in arteries without endothelium [17]. The benefit conferred by CD34+ cell delivery was seen after 28 days. It is also compatible with a paracrine hypothesis as outlined above, but the relative contribution of direct and indirect cell effects remain to be determined.

## Conclusion

The vasoprotective effects of freshly isolated human CD34+ cells without *in vitro *manipulation have been demonstrated in this novel animal model of carotid injury. Improvement in arterial vasoreactivity and decrease in neointima formation was observed in conjunction with delivery of selected CD34+ cells. This pre-clinical model has important implications for translational studies to clinical medicine.

## Competing interests

The authors declare that they have no competing interests.

## Authors' contributions

TK designed and performed the animal studies and analysis. BB designed and performed the animal studies and analysis. TW provided technical expertise for the animal studies. AD provided expertise and support for the cell isolation procedures. AL performed the vascular reactivity studies. RS provided the conceptual framework, designed the studies, and reviewed the analysis. The manuscript was written and approved by all members of the team.

## References

[B1] Gulati R, Jevremovic D, Peterson TE, Witt TA, Kleppe LS, Mueske CS, Lerman A, Vile RG, Simari RD (2003). Autologous culture-modified mononuclear cells confer vascular protection after arterial injury. Circulation.

[B2] Gulati R, Jevremovic D, Witt TA, Kleppe LS, Vile RG, Lerman A, Simari RD (2004). Modulation of the vascular response to injury by autologous blood-derived outgrowth endothelial cells. Am J Physiol Heart Circ Physiol.

[B3] Griese DP, Ehsan A, Melo LG, Kong D, Zhang L, Mann MJ, Pratt RE, Mulligan RC, Dzau VJ (2003). Isolation and transplantation of autologous circulating endothelial cells into denuded vessels and prosthetic grafts: implications for cell-based vascular therapy. Circulation.

[B4] Froehlich H, Gulati R, Boilson B, Witt T, Harbuzariu A, Kleppe L, Dietz AB, Lerman A, Simari RD (2009). Carotid Repair Using Autologous Adipose-Derived Endothelial Cells. Stroke.

[B5] Asahara T, Murohara T, Sullivan A, Silver M, Zee R van der, Li T, Witzenbichler B, Schatteman G, Isner J (1997). Isolation of putative progenitor endothelial cells for angiogensis. Science.

[B6] Mohle R (1997). Transendothelial migration of CD34+ and mature hematopoietic cells: an in vitro study using a human bone marrow endothelial cell line. Blood.

[B7] Dietz AB, Bulur PA, Emery RL, Winters JL, Epps DE, Zubair AC, Vuk-Pavlovic S (2006). A novel source of viable peripheral blood mononuclear cells from leukoreduction system chambers. Transfusion.

[B8] Urbich C, Dimmeler S (2004). Endothelial progenitor cells: characterization and role in vascular biology. Circ Res.

[B9] Assmus B, Schachinger V, Teupe C, Britten M, Lehmann R, Dobert N, Grunwald F, Aicher A, Urbich C, Martin H (2002). Transplantation of progenitor cells and regeneration enhancement in acute myocardial infarction (TOPCARE). Circulation.

[B10] Kalka C, Masuda H, Takahashi T, Kalka-Moll WM, Silver M, Kearney M, Li T, Isner JM, Asahara T (2000). Transplantation of ex vivo expanded endothelial progenitor cells for therapeutic neovascularization. PNAS.

[B11] Kawamoto A, Gwon H-C, Iwaguro H, Yamaguchi J-I, Uchida S, Masuda H, Silver M, Ma H, Kearney M, Isner J, Asahara T (2001). Therapeutic potential of ex vivo expanded endothelial progenitor cells for myocardial ischemia. Circulation.

[B12] Schatteman G, Hanlon H, Jiao C, Dodds S, Christy B (2000). Blood-derived angioblasts accelerate blood-flow restoration in diabetic mice. Journal of Clinical Investigation.

[B13] Kaushal S, Amiel GE, Guleserian KJ, Shapira OM, Perry T, Sutherland FW, Rabkin E, Moran AM, Schoen FJ, Atala A (2001). Functional small-diameter neovessels created using endothelial progenitor cells expanded ex vivo. Nat Med.

[B14] Schmeisser A, Garlichs CD, Zhang H, Eskafi S, Graffy C, Ludwig J, Strasser RH, Daniel WG (2001). Monocytes coexpress endothelial and macrophagocytic lineage markers and form cord-like structures in Matrigel and angiogenic conditions. Cardiovascular Research.

[B15] Kamihata H, Matsubara H, Nishiue T, Fujiyama S, Tsutsumi Y, Ozono R, Masaki H, Mori Y, Iba O, Tateishi E (2001). Implantation of bone marrow mononuclear cells into ischemic myocardium enhances collateral perfusion and regional function via side supply of angioblasts, angiogenic ligands, and cytokines. Circulation.

[B16] Kocher AA, Schuster MD, Szabolcs MJ, Takuma S, Burkhoff D, Wang J, Homma S, Edwards NM, Itescu S (2001). Neovascularization of ischemic myocardium by human bone-marrow-derived angioblasts prevents cardiomyocyte apoptosis, reduces remodeling and improves cardiac function. Nature Medicine.

[B17] Kullo I, Mozes G, Schwartz R, Gloviczki P, Crotty T, Barber D, Katusic Z, O'Brien T (1997). Adventitial gene transfer of recombinant endothelial nitric oxide synthase to rabbit carotid arteries alters vascular reactivity. Circulation.

